# Identification of GNG7 as a novel biomarker and potential therapeutic target for gastric cancer via bioinformatic analysis and *in vitro* experiments

**DOI:** 10.18632/aging.204545

**Published:** 2023-02-24

**Authors:** Houyu Duan, Biao Chen, Wei Wang, Hesheng Luo

**Affiliations:** 1 Department of Gastroenterology, Renmin Hospital of Wuhan University, Wuhan 430060, Hubei, P.R. China; 2 Department of Oncology, Renmin Hospital of Wuhan University, Wuhan 430060, Hubei, P.R. China; 3 Department of Hepatobiliary Surgery, Renmin Hospital of Wuhan University, Wuhan 430060, Hubei, P.R. China

**Keywords:** GNG7, gastric cancer, bioinformatic analysis, biomarker, therapeutic target

## Abstract

Gastric cancer (GC) is one of the most common malignancies with unfavorable prognoses. The present study aimed to identify novel biomarkers or potential therapeutic targets in GC via bioinformatic analysis and *in vitro* experiments. The Gene Expression Omnibus and The Cancer Genome Atlas databases were used to screen the differentially expressed genes (DEGs). After protein-protein interaction network construction, both module and prognostic analyses were performed to identify prognosis-related genes in GC. The expression patterns and functions of G protein γ subunit 7 (GNG7) in GC were then visualized in multiple databases and further verified using *in vitro* experiments. A total of 897 overlapping DEGs were detected and 20 hub genes were identified via systematic analysis. After accessing the prognostic value of the hub genes using the online server Kaplan-Meier plotter, a six-gene prognostic signature was identified, which was also significantly correlated with the process of immune infiltration in GC. The results of open-access database analyses suggested that GNG7 is downregulated in GC; this downregulation was associated with tumor progression. Furthermore, the functional enrichment analysis unveiled that the GNG7-coexpressed genes or gene sets were closely correlated with the proliferation and cell cycle processes of GC cells. Finally, *in vitro* experiments further confirmed that GNG7 overexpression inhibited GC cell proliferation, colony formation, and cell cycle progression and induced apoptosis. As a tumor suppressor gene, GNG7 suppressed the growth of GC cells via cell cycle blockade and apoptosis induction and thus may be used as a potential biomarker and therapeutic target for GC.

## INTRODUCTION

As per global statistics, gastric cancer (GC) ranked fourth in cancer-related deaths, causing >768,000 fatalities in 2020 [1]. Stomach adenocarcinoma (STAD) remains the most common pathological type of GC, which originates from the normal gastric mucosal epithelium [2]. Owing to the nonspecific symptoms at early stages, most patients with GC are diagnosed at advanced stages, leading to the loss of optimal surgical treatment opportunity [3]. Traditional strategies employed in the treatment of GC include surgery, chemotherapy, radiotherapy, biotherapy, and others; however, their overall therapeutic effect is far from satisfactory. Besides, the different levels of metastases and postoperative recurrence rates remain the predominant contributing factors in the invalidity or failure of clinical treatment [4]. The 5-year overall survival rate of patients with localized GC can reach 90%; however, the same rate in patients with progressive stages is usually <5% [5, 6]. Therefore, it is imperative for researchers to find novel biomarkers and effective targets for improving the early diagnosis rate and clinical outcomes of patients with GC.

Through high-throughput sequencing and gene microarray technologies, bioinformatic analysis has recently been increasingly used to search for molecular markers or targets involved in tumor development and progression [7–10]. In the present study, the Gene Expression Omnibus (GEO, http://www.ncbi.nlm.nih.gov/geo) and The Cancer Genome Atlas Stomach Adenocarcinoma (TCGA-STAD, https://portal.gdc.cancer.gov/) databases were used to screen the common differentially expressed genes (DEGs) between GC tissues and normal controls. In addition, after protein–protein interaction (PPI) network and module analyses, the online portal Kaplan–Meier plotter (http://www.kmplot.com/analysis/index.php?p=background) and Tumor Immune Estimation Resource (TIMER, https://cistrome.shinyapps.io/timer/) were utilized to access the prognostic value of the identified hub genes. Finally, the seldom reported G protein γ subunit 7 (GNG7) was selected to explore its expression patterns and potential roles in GC via several public databases and functional experiments *in vitro.* Via a comprehensive analysis, the prognostic implication and therapeutic potential of GNG7 in the progression of GC were discovered. This information may provide novel insights for future studies.

## MATERIALS AND METHODS

### Microarray data sources and preprocessing

The gene expression profile of the GSE65801 dataset, based on the GPL14550 Agilent-028004 SurePrint G3 Human GE 8x60K Microarray platform, was retrieved and downloaded from the GEO database; the dataset included 32 GC tissues and 32 normal gastric tissues. The STAD RNA-seq data, which contained 375 STAD tissues and 32 normal tissues, were downloaded from the TCGA portal. The background correction, log_2_ conversion, and quantile normalization of the raw data of the above two datasets were performed using the “affy” package in R 4.2.3 [11]. Probes without gene symbols or genes without available data were deleted. If numerous probes were annotated with a single gene, their median value was adopted as the gene expression level. However, if many genes were mapped to a single probe, the corresponding data were removed for the lack of specificity.

### DEG screening

The GEO2R (https://www.ncbi.nlm.nih.gov/geo/geo2r/) tool was applied for screening DEGs between GC and normal control samples in the GSE65801 dataset, whereas the “limma” R package was utilized for screening DEGs between GC and normal control samples in the TCGA-STAD dataset [12]. The adjusted *P*-value < 0.05 and |log_2_ fold change (FC)| > 1 were set as the cutoff criteria. The “ggplot2” and “pheatmap” R packages were applied to visualize the DEGs of each database by drawing volcano plots and heatmaps, respectively. These DEGs were subsequently classified into the up- and downregulated groups according to their log_2_FC value, and the overlapping up- or downregulated DEGs between the GSE65801 and TCGA-STAD datasets were analyzed using the Venny 2.1 tool (https://bioinfogp.cnb.csic.es/tools/venny/). Finally, the identified intersecting DEGs were further analyzed.

### Gene Ontology (GO) and Kyoto Encyclopedia of Genes and Genomes (KEGG) enrichment analyses of DEGs

As a well-known bioinformatics tool, GO annotates gene functions in three groups: biological process, cellular component, and molecular function [13]. KEGG is a widely used reference database to unravel the potential signaling pathways of the interaction network from a provided list of genes [14]. To evaluate the biological roles of the identified intersected DEGs, their GO and KEEG pathway enrichment analyses were processed via the Database for Annotation Visualization and Integrated Discovery (DAVID; http://david.ncifcrf.gov), which is an open-source website that helps comprehensively interpret the genomic information and biological information of a given gene or protein collection [15]. Furthermore, the results of the above enrichment analyses were visualized via the bubble charts plotted from an online bioinformatics platform (http://www.bioinformatics.com.cn). *P* < 0.05 was considered the cutoff of statistical significance.

### PPI network construction, module analysis, and candidate hub gene selection

The PPI network of the up- or downregulated overlapping DEGs was primarily constructed using the Search Tool for the Retrieval of Interacting Genes (STRING, http://string.embl.de/) database with the following cutoff standard: confidence score ≥ 0.4 and maximum interactors number = 0. The network was then visualized via the Cytoscape software (version 3.6.1) [16, 17]. In addition, the Molecular Complex Detection plug-in of Cytoscape was used to screen the most important module of the constructed PPI network [18]; the following default criteria were used for module analysis: degree cutoff value = 2, node score cutoff value = 0.2, K score = 2, and maximum depth = 100 [19]. Then, the DAVID database was utilized to detect the enriched KEGG pathways of the top three modules. Moreover, the three methods of node connect degree (Degree), maximal clique centrality (MCC), and maximal neighborhood component (MNC) in the CytoHubba plug-in of Cytoscape were applied for candidate hub gene selection. The genes scored in the top 20 of each method were chosen and employed for intersection analysis. The intersecting genes shared among all three methods were identified as the candidate hub genes, which were further visualized via the Venn diagram.

### Validation of candidate hub gene expression in Gene Expression Profiling Interactive Analysis (GEPIA)

GEPIA (http://gepia.cancer-pku.cn/) is a convenient online tool that is widely utilized in several customizable analyses, including differential expression analysis, correlation analysis, prognosis analysis, and others, based on the RNA-seq expression data of 9,736 tumor and 8,587 normal samples from TCGA and Genotype-Tissue Expression programs [20]. In the present study, GEPIA (version 2) was used to analyze expression differences in the candidate hub genes between GC and normal control samples.

### Survival analysis of hub genes

The Kaplan–Meier plotter is a web portal designed for performing survival analysis based on the gene expression and prognostic data of 21 cancer types derived from GEO, European Genome-phenome Archive, and TCGA databases [21]. In the present study, the prognostic values of the hub genes for the overall survival of patients with GC were estimated using the Kaplan–Meier plotter via the log-rank test. Besides, the median value of transcripts per million was adopted as the cutoff to dichotomize patients with GC into low- and high-expression groups, and log-rank *P* < 0.05 was considered statistically significant.

### Association between the expression of signature genes and infiltration levels of immune cells

TIMER (https://cistrome.shinyapps.io/timer/) is an online database resource that enables the analysis of the relationships of multiple factors, such as gene expression, mutation, and copy number alteration, with immune cell infiltration in different tumor types [22]. In the current study, TIMER was utilized to analyze the associations between the expression of signature genes and infiltration levels of B cells, CD4+ T cells, CD8+ T cells, macrophages, neutrophils, and dendritic cells in GC.

### Correlation and diagnostic value analysis of signature genes

To examine whether the signature genes are associated with each other, the “correlation analysis” module of GEPIA2 and “coexpression analysis” module of STRING were used to visualize the pairwise relationships among these genes. FunRich is a stand-alone bioinformatic analysis software that principally provides the functional enrichment and network analyses of the desired genes or proteins [23]. In the present study, FunRich (version 3.1.3) was used to unravel the potential interactions between the signature genes and their related genes. GeneMANIA (http://www.genemania.org), a versatile online server, is capable of performing the interaction, coexpression, and functional enrichment analyses of the given gene lists based on abundant genomic data [24]. The present study utilized GeneMANIA to annotate the functions of the signature genes and their coexpression genes. To further appraise the diagnostic sensitivity and specificity of the signature genes, their receiver operating characteristic curves were plotted using the bioinformatics platform via the gene expression profiles derived from the TCGA-STAD dataset. Besides, the signature genes with the area under the curve of >0.9 were considered as having high diagnostic accuracy.

### Expression pattern and clinicopathological significance of GNG7 in GC

Among the signature genes, the rarely reported GNG7 was selected to explore its potential roles in GC progression. TIMER was primarily utilized to examine the expression patterns of GNG7 in 32 cancer types from the TCGA database. As an online datamining platform, Oncomine (http://www.oncomine.org) integrates data from 715 datasets with >80,000 samples for genome-wide bioinformatic analysis [25]. In the present study, Oncomine was used to detect the expression levels of GNG7 in diverse cancers with *P* value < 1E-4 and |log_2_FC| > 2 as the threshold. The GEPIA2 database was further used to identify the expression levels of GNG7 in multiple cancers. Hiplot (https://hiplot.org), an interactive web service, helps users analyze and visualize biomedical data [26]. Using the Violin-Chart tool in Hiplot, the differential expression of GNG7 in GC and normal controls was detected and validated in the GSE13861 (71 GC and 19 normal tissues), GSE13911 (38 GC and 31 normal tissues), and GSE19826 (12 GC and 12 normal tissues) datasets, which were downloaded from the GEO database. UALCAN (http://ualcan.path.uab.edu/index.html) is an open-access web-portal that helps perform the in-depth analysis of cancer-associated omics data from the TCGA, MET500, and CPTAC databases [27]. In the current study, the “TCGA analysis” module of UALCAN was used to explore the expression of GNG7 and its association with the clinicopathological features (sex, age, race, tumor grade, histological subtype, nodal metastasis status, tumor stage, and others) of patients with GC.

### Enrichment analysis of GNG7-coexpressed genes in GC

LinkedOmics (http://www.LinkedOmics.org/login.php), a flexible online database resource, enables the analysis of multiple omics data across 32 cancer types from the TCGA database [28]. After querying the “LinkFinder” module of LinkedOmics using the Pearson correlation test, the volcano plots and heatmaps of genes coexpressed with GNG7 in the TCGA-STAD cohort were obtained. Metascape (http://metascape.org/) is an open-access web server that offers the gene annotation and analysis of a given list of genes [29]. In the current study, Metascape was used to analyze the enrichment analysis of the biological processes and pathways of GNG7-coexpressed genes. The biological pathways enriched in these genes were further interpreted using FunRich 3.1.3.

### Gene set enrichment analysis (GSEA)

GSEA is a powerful algorithm for predicting potential biological processes or signaling pathways correlated with the specific gene set by comparing the statistical differences of enrichment analysis between the two groups previously defined via the phenotypes of the desired genes [30]. In the current study, the samples from the TCGA-STAD dataset were classified into GNG7 low- and high-expression groups according to the median expression value, and GSEA (version 4.2.3) software (http://software.broadinstitute.org/gsea/index.jsp) was then used to speculate the signaling pathways associated with GNG7 expression in GC. c2.cp.kegg.v7.5.1. symbols.gmt, downloaded from the Molecular Signature Database (http://www.gseamsigdb.org/gsea/msigdb/), was used as the reference gene set, and |normalized enrichment score | > 1, nominal *P* < 0.05 and FDR < 0.25 were used as the cutoff criteria.

### Cell lines and cell transfection

The human gastric epithelial cell line GES-1 and GC cell lines (AGS, BGC823, HGC27, and SGC7901) were cultured in Roswell Park Memorial Institute-1640 medium (Jinuo, Hangzhou, China) supplemented with 10% fetal bovine serum (Evergreen, Hangzhou, China) and 1% penicillin/streptomycin (Jinuo, Hangzhou, China). The cells were incubated at 37° C and 5% CO_2_.

SGC7901 cells were transfected with the human pcDNA3.1-GNG7 or pcDNA3.1 plasmid (GenePharma, Suzhou, China) using Lipofectamine 2000 (Invitrogen, MA, USA) according to the manufacturer’s instructions. After 72 h, the transfected cells were collected for the subsequent experiments.

### RNA extraction and quantitative reverse transcription-polymerase chain reaction (qRT-PCR)

The total RNA of cells was extracted using TRIzol (Invitrogen, MA, USA) and then used to synthesize cDNA via PrimeScript™ RT reagent kit (TaKaRa, Kyoto, Japan). The synthesized cDNA was used to perform qRT-PCR with SYBR^®^ Premix Ex Taq™ II (TaKaRa, Kyoto, Japan) in the CFX Maestro Detection System (Bio-Rad, CA, USA) according to the manufacturer’s instructions. The amplification conditions were 95° C for 30 s, 95° C for 5 s, and 60° C for 30 s for a total of 40 cycles. The primers (Tsingke, Wuhan, China) used for PCR were shown in Table 1. Glyceraldehyde 3-phosphate dehydrogenase was adopted as the internal reference. The relative expression levels of genes in different cells were analyzed using the 2^−ΔΔCt^ method [31].

**Table 1:** The primer sequences used in qRT-PCR.

**Genes**	**Sequence (5′→3′)**
GNG7	Forward: 5′-CAGCCACTA ACAACATAGCCCAGG-3′
	Reverse: 5′-TGTCCTT AAAGGGGTTCTCCGAGG-3′
RASGRP2	Forward: 5′-TCCACATCTACCAACAATCC-3′
	Reverse: 5′-CCTTCAGCTCCTTGATCTG-3′
AFF3	Forward: 5′-ACTCAACAGGATGATGGC-3′
	Reverse: 5′-TGCCTAAAGTGTTCTGGATC-3′
NAP1L2	Forward: 5′-CAGACCGTCCAAAAGGACTTA-3′
	Reverse: 5′-AGTAAGGGTTGGTACATTTCA-3′
ANLN	Forward: 5′-CAAGATGTATCCAATGACT-3′
	Reverse: 5′-TGACTGAAGAATGAATGTT-3′
MELK	Forward: 5′-GCTGCAAGGTATAATTGATGGA-3′
	Reverse: 5′-CAGTAACATAATGACAGATGGGC-3′
DEPDC1	Forward: 5′-CCGAACATAGAAGGACAA-3′
	Reverse: 5′-CTCTTGGTCTTGAACAGT-3′
GAPDH	Forward: 5′-GGAGTCCACTGGCGTCTTCA-3′
	Reverse: 5′-GTCATGAGTCCTTCCACGATACC-3′

Abbreviations: qRT-PCR, RNA extraction and quantitative reverse transcription-polymerase chain reaction.

### Proliferation assay

The transfected cells were inoculated into 96-well plates and cultured in an incubator (5% CO_2_ and 37° C). After incubation for 0, 24, 48, and 72 h, the proliferation abilities of the treated cells were determined using the CCK-8 assay. At each detection time point, the cells were treated with 10 μL of CCK-8 regent (Biosharp, Beijing, China) per well and cultured for another 2 h. The absorbance was measured using a microplate reader (Bio-Rad, CA, USA) at the wavelength of 450 nm.

### Colony-formation assay

The transfected cells were inoculated into a 6-well plate (500 cells/well) and cultured with complete medium in an incubator (5% CO_2_ and 37° C). The medium was replaced every 2~3 days. After incubation for 14 days, the formed colonies, each containing >50 cells, were fixed with 4% paraformaldehyde and stained with 0.1% crystal violet. The number of colonies in each well was then counted and photographed.

### Cell cycle and apoptosis assay

After transfection, the cells were collected and fixed with 70% ethanol and stored in a cryogenic refrigerator (−20° C) overnight. Next day, the cells were washed twice with PBS and stained with propidium iodide/RNase staining buffer (BD Biosciences, CA, USA) for 15 min in the dark at room temperature. For the apoptosis assay, the cells were double-stained with Annexin V PE and 7-AAD using the Annexin V PE apoptosis detection kit I (BD Biosciences, CA, USA) according to the manufacturer’s instructions. FACSCalibur™ flow cytometer (BD Biosciences, CA, USA) was used to determine the cell cycle and apoptotic rate of the stained cells.

### Statistical analyses

SPSS 23.0 (SPSS Inc., IL, USA) and GraphPad Prism 8.01 (GraphPad Software Inc., CA, USA) were used for data analyses. Continuous data obtained from three separate experiments are depicted as mean ± standard deviation. Student’s t-test was used to compare two groups, whereas one-way ANOVA was applied to compare multiple groups. The two-tailed *P*-value of <0.05 was regarded as statistically significant.

## RESULTS

### DEG identification

**Figure 1:**
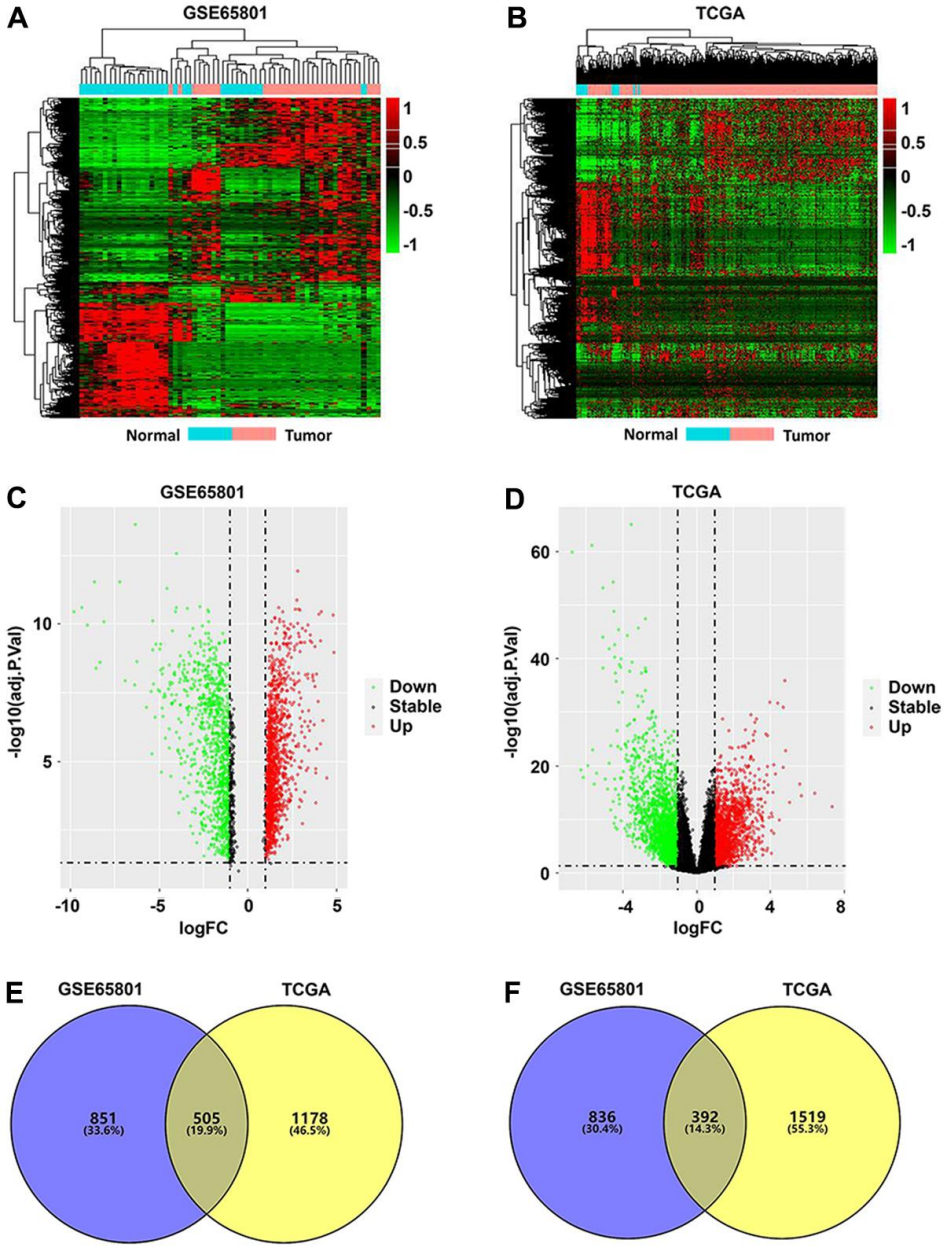
**Identification of differentially expressed genes (DEGs) in GC.** (**A**, **B**) DEG heatmaps of GC and normal tissues from the GSE65801 (**A**) and TCGA (**B**) datasets. (**C**, **D**) DEG volcano plots of GC and normal tissues from the GSE65801 (**C**) and TCGA (**D**) datasets. (**E**, **F**) Venn diagram showing the up- (**E**) and downregulated overlapping DEGs (**F**) between the GSE65801 and TCGA datasets. DEGs, differentially expressed genes; FC, fold change; GC, gastric cancer; and TCGA, The Cancer Genome Atlas.

A total of 2,584 DEGs were identified from the GSE65801 dataset, including 1,356 upregulated and 1,228 downregulated DEGs, whereas 3,594 DEGs were screened from the TCGA-STAD dataset, which included 1,683 upregulated and 1,911 downregulated DEGs. The DEGs identified from the two datasets were visualized via heatmaps and volcano plots (Figure 1A–1D). Venn analysis revealed a total of 505 upregulated (Figure 1E) and 392 downregulated intersecting DEGs (Figure 1F) between the GSE65801 and TCGA-STAD datasets.

### GO and KEGG pathway analyses of DEGs

**Figure 2:**
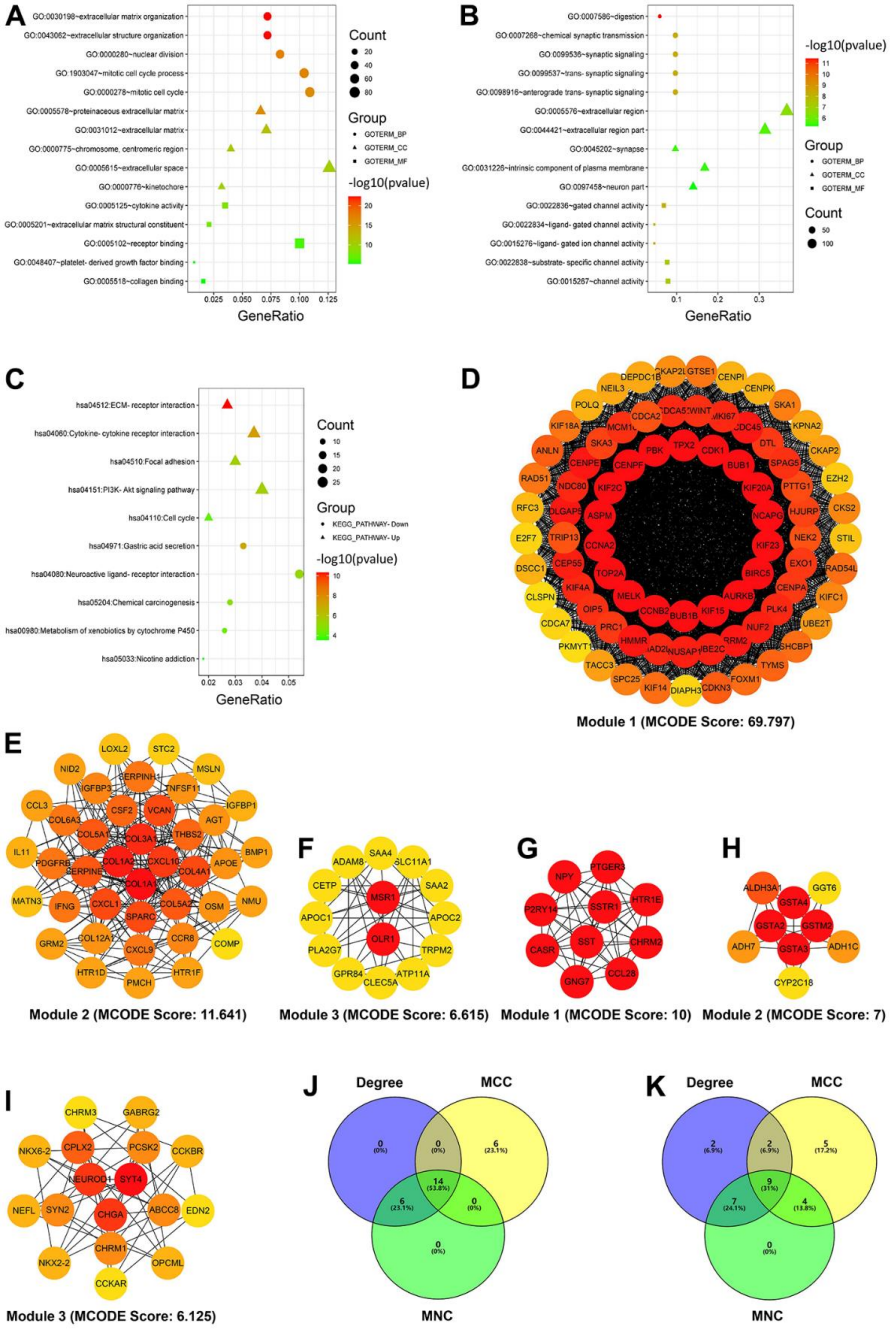
**Enrichment and module analyses of differentially expressed genes (DEGs).** (**A**) GO enrichment analysis of the upregulated DEGs. (**B**) GO enrichment analysis of the downregulated DEGs. (**C**) KEGG pathway enrichment analysis of the up- and downregulated DEGs. (**D**–**F**) Top three modules of the PPI network from the upregulated DEGs (the color intensity of circles represents the connectivity degree of DEGs). (**G**–**I**) Top three modules of the PPI network from the downregulated DEGs (the color intensity of circles represents the connectivity degree of DEGs). (**J**, **K**) Three algorithms to screen the up- (**J**) and downregulated candidate hub genes (**K**) using the Venn diagram. Degree, node connect degree; DEGs, differentially expressed genes; GO, Gene Ontology; KEGG, Kyoto Encyclopedia of Genes and Genomes; MCC, maximal clique centrality; MNC, maximal neighborhood component; and PPI, protein–protein interaction.

For a better understanding of the up and downregulated intersecting DEGs, the two gene lists were mapped into the DAVID database for GO and KEGG pathway enrichment analyses. The GO enrichment analysis of the biological process revealed that the upregulated DEGs were associated with extracellular matrix organization, extracellular structure organization, nuclear division, mitotic cell cycle process, and mitotic cell cycle, whereas the downregulated DEGs were involved in the processes of digestion, chemical synaptic transmission, synaptic signaling, trans-synaptic signaling, and anterograde trans-synaptic signaling. Cellular component enrichment analysis revealed that the upregulated DEGs were predominantly enriched in proteinaceous extracellular matrix, extracellular matrix, chromosome centromeric region, extracellular space, and kinetochore, whereas the downregulated DEGs were enriched in the extracellular region, extracellular region part, synapse, intrinsic component of the plasma membrane, and neuron part. Regarding the molecular function, the upregulated DEGs were principally associated with cytokine activity, extracellular matrix structural constituent, receptor binding, platelet-derived growth factor binding, and collagen binding, whereas the downregulated DEGs were correlated with gated channel activity, ligand-gated channel activity, ligand-gated ion channel activity, substrate-specific channel activity, and channel activity (Figure 2 and Table 2).

**Table 2:** GO analysis of DEGs in GC.

**Expression**	**Category**	**Term**	**Count(%)**	***P* value**	**FRD**
Upregulated	GOTERM_BP_FAT	GO:0030198~extracellular matrix organization	51(7.20)	3.99E-23	1.19E-19
	GOTERM_BP_FAT	GO:0043062~extracellular structure organization	51(7.20)	4.57E-23	1.19E-19
	GOTERM_BP_FAT	GO:0000280~nuclear division	59(8.32)	7.39E-18	1.28E-14
	GOTERM_BP_FAT	GO:1903047~mitotic cell cycle process	74(10.44)	1.37E-17	1.78E-14
	GOTERM_BP_FAT	GO:0000278~mitotic cell cycle	77(10.86)	3.12E-17	3.14E-14
	GOTERM_CC_FAT	GO:0005578~proteinaceous extracellular matrix	47(6.63)	5.09E-17	2.47E-14
	GOTERM_CC_FAT	GO:0031012~extracellular matrix	50(7.05)	1.83E-12	4.44E-10
	GOTERM_CC_FAT	GO:0000775~chromosome centromeric region	28(3.95)	1.43E-11	2.30E-09
	GOTERM_CC_FAT	GO:0005615~extracellular space	89(12.56)	5.00E-11	5.19E-09
	GOTERM_CC_FAT	GO:0000776~kinetochore	23(3.25)	5.35E-11	5.19E-09
	GOTERM_MF_FAT	GO:0005125~cytokine activity	25(3.53)	6.23E-09	5.73E-06
	GOTERM_MF_FAT	GO:0005201~extracellular matrix structural constituent	15(2.12)	3.14E-08	1.44E-05
	GOTERM_MF_FAT	GO:0005102~receptor binding	71(10.02)	9.23E-07	2.83E-04
	GOTERM_MF_FAT	GO:0048407~platelet-derived growth factor binding	6(0.85)	5.34E-06	1.23E-03
	GOTERM_MF_FAT	GO:0005518~collagen binding	11(1.55)	6.86E-06	1.26E-03
Downregulated	GOTERM_BP_FAT	GO:0007586~digestion	23(5.87)	3.69E-12	1.49E-08
	GOTERM_BP_FAT	GO:0007268~chemical synaptic transmission	38(9.69)	4.87E-09	3.93E-06
	GOTERM_BP_FAT	GO:0099536~synaptic signaling	38(9.69)	4.87E-09	3.93E-06
	GOTERM_BP_FAT	GO:0099537~trans-synaptic signaling	38(9.69)	4.87E-09	3.93E-06
	GOTERM_BP_FAT	GO:0098916~anterograde trans-synaptic signaling	38(9.69)	4.87E-09	3.93E-06
	GOTERM_CC_FAT	GO:0005576~extracellular region	144(36.74)	3.11E-07	1.10E-04
	GOTERM_CC_FAT	GO:0044421~extracellular region part	123(31.38)	2.02E-06	3.11E-04
	GOTERM_CC_FAT	GO:0045202~synapse	38(9.69)	3.17E-06	3.11E-04
	GOTERM_CC_FAT	GO:0031226~intrinsic component of plasma membrane	66(16.84)	3.53E-06	3.11E-04
	GOTERM_CC_FAT	GO:0097458~neuron part	55(14.03)	5.22E-06	3.60E-04
	GOTERM_MF_FAT	GO:0022836~gated channel activity	27(6.89)	5.79E-09	2.05E-06
	GOTERM_MF_FAT	GO:0022834~ligand-gated channel activity	18(4.59)	7.04E-09	2.05E-06
	GOTERM_MF_FAT	GO:0015276~ligand-gated ion channel activity	18(4.59)	7.04E-09	2.05E-06
	GOTERM_MF_FAT	GO:0022838~substrate-specific channel activity	30(7.65)	4.05E-08	8.21E-06
	GOTERM_MF_FAT	GO:0015267~channel activity	31(7.91)	5.39E-08	8.21E-06

Abbreviations: BP, biological process; CC, cellular component; DEGs, differentially expressed genes; FDR, false discovery rate; GC, gastric cancer; GO, Gene Ontology; MF, molecular function.

KEGG pathway enrichment analysis results revealed that the upregulated DEGs were predominantly enriched in extracellular matrix–receptor interaction, cytokine–cytokine receptor interaction, focal adhesion, PI3K-Akt signaling pathway, and cell cycle, whereas the downregulated DEGs were primarily associated with gastric acid secretion, neuroactive ligand–receptor interaction, chemical carcinogenesis, xenobiotic metabolism via cytochrome P450, and nicotine addiction (Figure 2 and Table 3).

**Table 3:** KEGG pathway analysis of DEGs in GC.

**Expression**	**Term**	**Count(%)**	**Genes**	***P* value**	**FRD**
Upregulated	hsa04512:ECM-receptor interaction	19(2.68)	COL27A1, ITGA2, FN1, LAMC2, HMMR, THBS2, COL1A1, COMP, COL3A1, COL1A2, COL5A1, IBSP, COL4A1, COL5A3, COL5A2, ITGA11, SPP1, COL6A3, ITGB8	4.73E-11	9.23E-09
	hsa04060:Cytokine-cytokine receptor interaction	26(3.67)	CXCL9, TNFRSF6B, CSF2, CCL3L3, IL24, CXCL1, TNFRSF11B, CCL7, CCL3, CCR8, TNFSF11, CCL18, CCL15, IL11, TNFRSF12A, TNFRSF9, LIF, OSM, INHBA, OSMR, CXCL10, IFNG, IL2RA, TNFSF4, TNFSF9, TNFRSF25	4.13E-08	4.03E-06
	hsa04510:Focal adhesion	21(2.96)	PDGFRB, COL27A1, ITGA2, FN1, LAMC2, THBS2, PGF, VAV2, COL1A1, COMP, COL3A1, COL1A2, COL5A1, IBSP, COL4A1, COL5A3, COL5A2, ITGA11, SPP1, COL6A3, ITGB8	2.57E-06	1.46E-04
	hsa04151:PI3K-Akt signaling pathway	28(3.95)	LAMC2, THBS2, COMP, IBSP, MYB, SPP1, ITGB8, PDGFRB, ANGPT2, COL27A1, ITGA2, F2R, OSM, FN1, OSMR, PGF, COL1A1, COL3A1, EFNA3, COL1A2, COL5A1, COL4A1, COL5A3, IL2RA, COL5A2, ITGA11, COL6A3, PIK3AP1	3.00E-06	1.46E-04
	hsa04110:Cell cycle	14(1.98)	CDKN2A, BUB1B, PKMYT1, CDC25B, CCNA2, CCNB2, RBL1, CDC45, PTTG1, E2F1, CDK1, E2F3, BUB1, MAD2L1	7.08E-05	2.76E-03
Downregulated	hsa04971:Gastric acid secretion	13(3.32)	CAMK2B, CHRM3, KCNE2, KCNK10, KCNJ15, KCNJ16, ATP4B, ATP4A, SLC9A4, CCKBR, CA2, SST, KCNK2	9.59E-08	1.61E-05
	hsa04080:Neuroactive ligand-receptor interaction	21(5.36)	GABRB3, CHRM2, CHRM3, GRIA2, GABRA1, CHRM1, THRB, HTR1E, P2RY14, CHRNA7, PTGER3, SCTR, GRIK1, SSTR1, GABRG2, CCKAR, CCKBR, ADRB3, P2RX2, GRIA3, GRIA4	6.17E-06	5.19E-04
	hsa05204:Chemical carcinogenesis	11(2.81)	CBR1, ALDH3A1, GSTM2, GSTA4, ADH1C, GSTA3, CHRNA7, GSTA2, ADH7, CYP2C18, SULT2A1	1.50E-05	8.40E-04
	hsa00980:Metabolism of xenobiotics by cytochrome P450	10(2.55)	CBR1, ALDH3A1, GSTM2, GSTA4, ADH1C, GSTA3, GSTA2, AKR1C1, ADH7, SULT2A1	5.06E-05	2.13E-03
	hsa05033:Nicotine addiction	7(1.79)	GABRB3, GABRA1, GRIA2, CHRNA7, GABRG2, GRIA3, GRIA4	2.97E-04	9.99E-03

Abbreviations: Akt, protein kinase B; DEGs, differentially expressed genes; ECM, extracellular matrix; FDR, false discovery rate; GC, gastric cancer; KEGG, Kyoto Encyclopedia of Genes and Genomes; PI3K, phosphatidylinositol 3-kinase.

### PPI network construction, module analysis, and candidate hub gene selection

After searching the STRING database, the PPI network for the upregulated intersecting DEGs was first established using the Cytoscape software; the network contained 436 nodes and 4,895 edges (Supplementary Figure 1). The top three modules of this network (Figure 2D–2F) were then selected via module analysis using the Molecular Complex Detection plug-in of Cytoscape. KEGG enrichment analysis suggested that these three modules were significantly enriched in cell cycle, extracellular matrix–receptor interaction, and phagosome (Table 4). The top 20-ranked genes identified by all three algorithms (Degree, MCC, and MNC) in the CytoHubba plug-in were chosen as candidate hub genes. The identified upregulated candidate hub genes were as follows: CDK1, CCNA2, AURKB, BUB1B, TOP2A, BIRC5, CCNB2, BUB1, CENPF, KIF2C, PBK, NCAPG, TPX2, and KIF15 (Figure 2J and Table 5). The PPI network of the downregulated overlapping DEGs comprised 316 nodes and 736 edges (Supplementary Figure 2), and the top three modules of the network (Figure 2G–2I) were predominantly enriched in cAMP signaling pathway, chemical carcinogenesis, and neuroactive ligand–receptor interaction (Table 3). The identified downregulated candidate hub genes were as follows: SST, GCG, CHGA, GNG7, NPY, GHRL, CASR, NEUROD1, and CCKBR (Figure 2K and Table 5).

**Table 4:** KEGG pathway analysis of top 3 modules.

**Expression**	**Module**	**Term**	**Count(%)**	**Genes**	***P* value**	**FRD**
Upregulated	Module 1	hsa04110: Cell cycle	9(11.25)	CCNA2, CCNB2, CDC45, PTTG1, CDK1, BUB1B, PKMYT1, BUB1, MAD2L1	2.04E-08	6.74E-07
		hsa04914: Progesterone-med- iated oocyte maturation	6(7.5)	CCNA2, CCNB2, CDK1, PKMYT1, BUB1, MAD2L1	2.25E-05	3.72E-04
		hsa04114: Oocyte meiosis	6(7.5)	CCNB2, PTTG1, CDK1, PKMYT1, BUB1, MAD2L1	7.30E-05	8.04E-04
		hsa04115: p53 signaling pathway	4(5)	CCNB2, RRM2, CDK1, GTSE1	2.43E-03	2.01E-02
		hsa03430: Mismatch repair	2(2.5)	RFC3, EXO1	8.97E-02	0.59
	Module 2	hsa04512: ECM-receptor interaction	9(22.5)	COL1A1, COMP, COL3A1, COL1A2, COL5A1, COL4A1, COL5A2, COL6A3, THBS2	1.19E-09	7.76E-08
		hsa04974: Protein digestion and absorption	8(20)	COL1A1, COL3A1, COL1A2, COL5A1, COL4A1, COL12A1, COL5A2, COL6A3	4.19E-08	1.36E-06
		hsa04510: Focal adhesion	10(25)	PDGFRB, COL1A1, COMP, COL3A1, COL1A2, COL5A1, COL4A1, COL5A2, COL6A3, THBS2	6.86E-08	1.49E-06
		hsa05146: Amoebiasis	8(20)	COL1A1, COL3A1, CSF2, COL1A2, IFNG, COL5A1, COL4A1, COL5A2	1.53E-07	2.49E-06
		hsa04060: Cytokine-cytokine receptor interaction	10(25)	IL11, CXCL10, CXCL9, CSF2, IFNG, OSM, CCL3, CCR8, TNFSF11, CXCL1	2.84E-07	3.69E-06
	Module 3	hsa04145: Phagosome	2(14.29)	MSR1, OLR1	8.44E-02	0.59
Downregulated	Module 1	hsa04024: cAMP signaling pathway	5(50)	CHRM2, HTR1E, NPY, PTGER3, SSTR1	4.26E-05	9.37E-04
		hsa04080: Neuroactive ligand-receptor interaction	5(50)	CHRM2, HTR1E, P2RY14, PTGER3, SSTR1	1.58E-04	1.74E-03
		hsa04923: Regulation of lipolysis in adipocytes	2(20)	NPY, PTGER3	6.33E-02	0.46
	Module 2	hsa05204: Chemical carcinogenesis	8(88.89)	ALDH3A1, GSTM2, GSTA4, ADH1C, GSTA3, GSTA2, ADH7, CYP2C18	1.75E-13	2.62E-12
		hsa00982: Drug metabolism-cytochrome P450	7(77.78)	ALDH3A1, GSTM2, GSTA4, ADH1C, GSTA3, GSTA2, ADH7	2.05E-11	1.54E-10
		hsa00980: Metabolism of xenobiotics by cytochrome P450	7(77.78)	ALDH3A1, GSTM2, GSTA4, ADH1C, GSTA3, GSTA2, ADH7	3.47E-11	1.74E-10
		hsa00480: Glutathione metabolism	5(55.56)	GSTM2, GGT6, GSTA4, GSTA3, GSTA2	1.84E-07	6.88E-07
		hsa00350: Tyrosine metabolism	3(33.33)	ALDH3A1, ADH1C, ADH7	6.91E-04	2.07E-03
	Module 3	hsa04080: Neuroactive ligand-receptor interaction	5(29.41)	CHRM3, CCKAR, CHRM1, CCKBR, GABRG2	2.76E-04	4.42E-03
		hsa04020: Calcium signaling pathway	4(23.53)	CHRM3, CCKAR, CHRM1, CCKBR	1.30E-03	1.04E-02
		hsa04911: Insulin secretion	3(17.65)	CHRM3, CCKAR, ABCC8	5.13E-03	2.74E-02
		hsa04950: Maturity onset diabetes of the young	2(11.76)	NEUROD1, NKX2-2	3.35E-02	0.13
		hsa04971: Gastric acid secretion	2(11.76)	CHRM3, CCKBR	9.16E-02	0.29

Abbreviations: cAMP, cyclic adenosine monophosphate; ECM, extracellular matrix; KEGG, Kyoto Encyclopedia of Genes and Genomes.

**Table 5:** Up- and down-regulated candidate hub genes identified in PPI networks by different ranked methods in CytoHubba.

**Up-regulated**			**Down-regulated**		
**Degree**	**MCC**	**MNC**	**Degree**	**MCC**	**MNC**
***CDK1***	***NCAPG***	***CDK1***	***SST***	***SST***	***SST***
***CCNA2***	KIF23	***CCNA2***	GRIA2	***NPY***	***GCG***
***AURKB***	***BIRC5***	***AURKB***	***GCG***	CCL28	GRIA2
***BUB1B***	***AURKB***	***BUB1B***	***CHGA***	***GNG7***	***CHGA***
***TOP2A***	***KIF15***	***BIRC5***	SYT4	***CASR***	***NPY***
***BIRC5***	***BUB1B***	***CCNB2***	***GNG7***	SSTR1	GABRA1
***CCNB2***	***CCNB2***	***TOP2A***	GABRG2	CHRM2	SYT4
***BUB1***	MELK	***BUB1***	***NPY***	HTR1E	***GHRL***
UBE2C	***TOP2A***	UBE2C	GABRA1	PTGER3	GABRG2
CDC45	***CCNA2***	CDC45	***GHRL***	P2RY14	***GNG7***
RRM2	ASPM	RRM2	OPCML	***GCG***	OPCML
***CENPF***	***KIF2C***	***CENPF***	***CASR***	***GHRL***	CPLX2
***KIF2C***	***CENPF***	***KIF2C***	CPLX2	***CCKBR***	***CCKBR***
***PBK***	***PBK***	***PBK***	***NEUROD1***	CCKAR	***CASR***
***NCAPG***	***TPX2***	***NCAPG***	AQP4	***CHGA***	***NEUROD1***
***TPX2***	***CDK1***	***TPX2***	***CCKBR***	PCSK2	NEFL
DLGAP5	***BUB1***	DLGAP5	CHRM2	NKX2-2	SSTR1
CENPE	KIF20A	CENPE	CHRM1	NKX6-2	CCL28
MKI67	HMMR	MKI67	NEFL	***NEUROD1***	PCSK2
***KIF15***	CEP55	***KIF15***	NCAM1	CHRM1	NKX2-2

Abbreviations: MCC, maximal clique centrality; MNC, maximal neighborhood component; PPI, protein-protein interaction. Gene symbols with bold and italic were the top 20 intersection genes by three ranked methods.

### Validation of candidate hub gene expression in GEPIA

**Figure 3:**
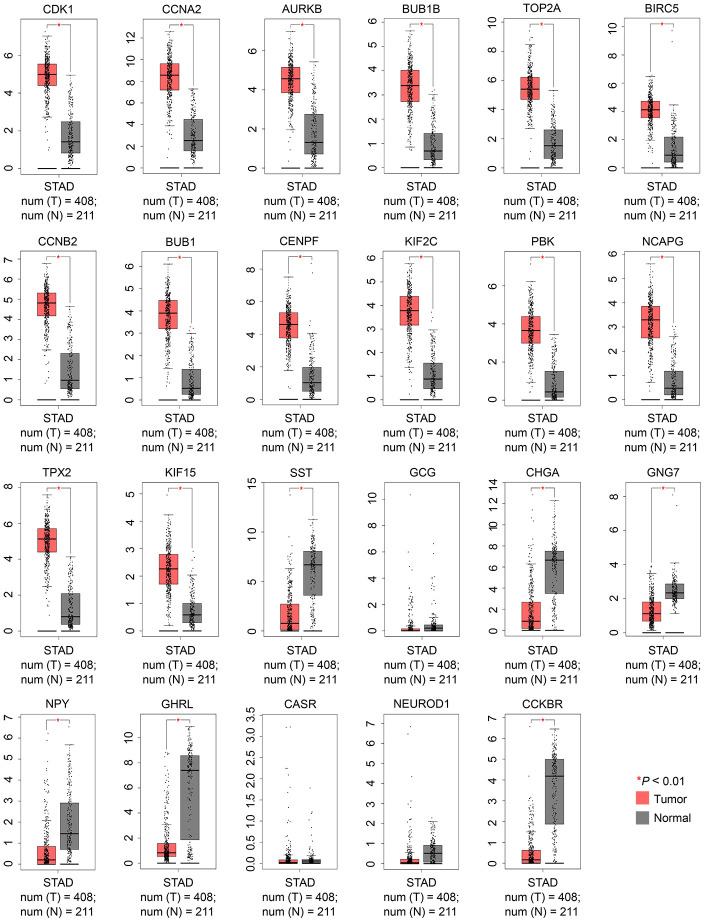
**Validation of the expression levels of 23 candidate hub genes between GC and normal controls using the GEPIA2 database.** |Log_2_FC|: 1 and *P*-value: 0.01 were set as the cutoff. FC, fold change; GC, gastric cancer; GEPIA, Gene Expression Profiling Interactive Analysis. N, normal; STAD, stomach adenocarcinoma; and T, tumor.

To confirm whether the 23 candidate hub genes were reliable, the “Expression DIY” module of GEPIA2 was utilized to determine their expression levels in GC. All candidate hub genes identified via the PPI network of upregulated DEGs showed increased expression levels in GC tissues compared with normal tissues, whereas the candidate hub genes obtained from the PPI network of downregulated DEGs had significantly decreased expression levels in GC compared with normal controls, except for CASR, GCG, and NEUROD1 (Figure 3). Because the expression trends of the 14 upregulated genes (CDK1, CCNA2, AURKB, BUB1B, TOP2A, BIRC5, CCNB2, BUB1, CENPF, KIF2C, PBK, NCAPG, TPX2, and KIF15) and 6 downregulated genes (SST, CHGA, GNG7, NPY, GHRL, and CCKBR) were in accordance with the study data, they were selected as the real hub genes and further analyzed.

### Prognostic value analysis of hub genes

**Figure 4:**
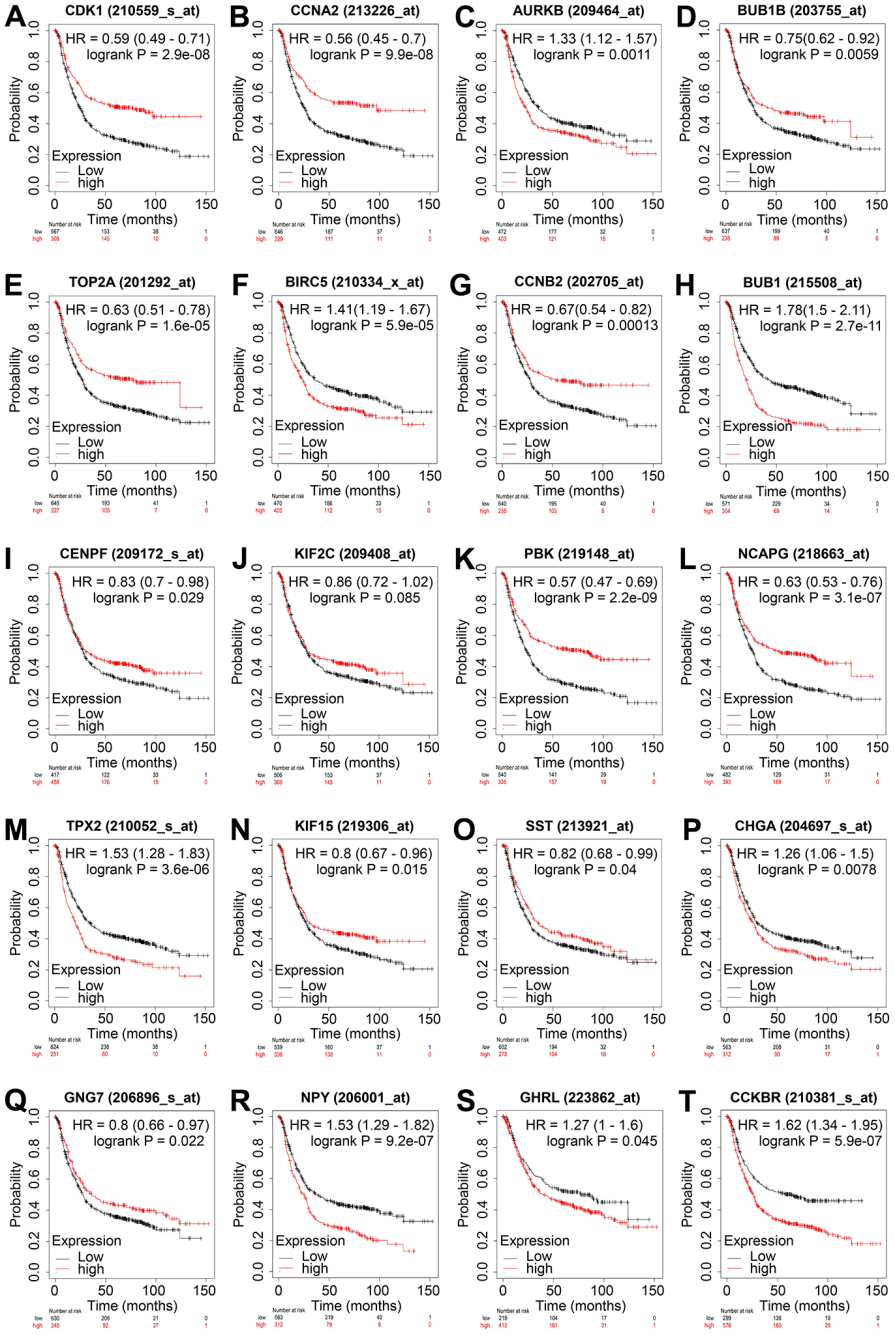
**Overall survival analysis of the 20 hub genes in GC.** Overall survival of patients with GC with low and high levels of CDK1 (**A**), CCNA2 (**B**), AURKB (**C**), BUB1B (**D**), TOP2A (**E**), BIRC5 (**F**), CCNB2 (**G**), BUB1 (**H**), CENPF (**I**), KIF2C (**J**), PBK (**K**), NCAPG (**L**), TPX2 (**M**), KIF15 (**N**), SST (**O**), CHGA (**P**), GNG7 (**Q**), NPY (**R**), GHRL (**S**), and CCKBR (**T**) are displayed using Kaplan–Meier curves. Log-rank *P* < 0.05 was regarded as statistically significant. GC, gastric cancer; HR, hazard ratio.

To determine the prognostic value of the 20 hub genes, the Kaplan–Meier plotter was used to perform the overall survival analysis. The results revealed that the mRNA expression of all 14 upregulated hub genes was associated with the overall survival of patients with GC. Among the genes, patients with higher expression levels of AURKB, BIRC5, BUB1, and TPX2 had shorter overall survival compared with the control groups, which is consistent with our speculation that the increased expression of these genes would reduce the overall survival of patients (Figure 4). The expression levels of all six downregulated hub genes were also associated with the overall survival of patients with GC; however, compared with controls, only the low expression of GNG7 and SST was associated with the unfavorable overall survival of patients with GC, again consistent with our aforementioned speculation (Figure 4). Therefore, a six-gene signature, including four upregulated genes AURKB, BIRC5, BUB1, and TPX2 and two downregulated genes SST and GNG7, was identified as an indicator for GC.

### Association between the expression of signature genes and infiltration levels of immune cells

**Figure 5:**
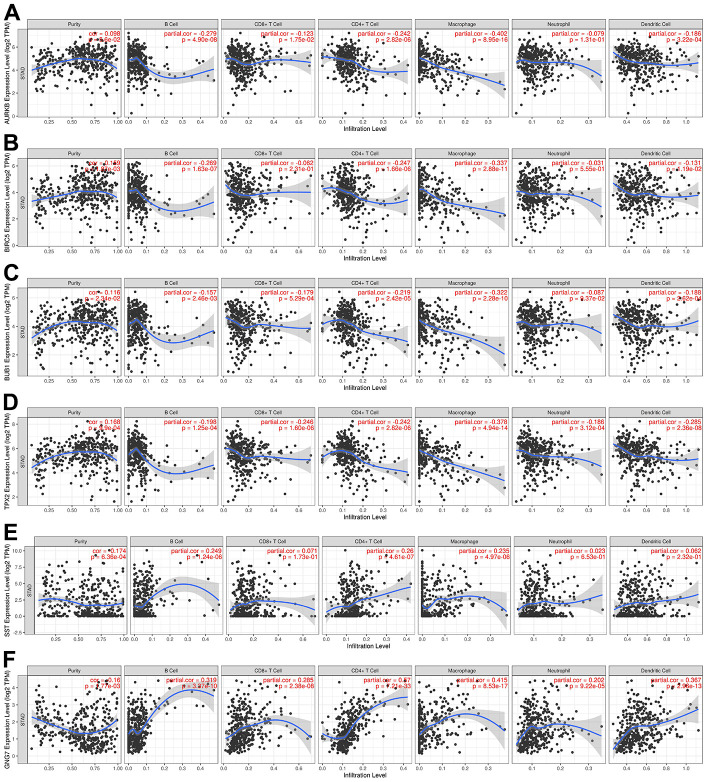
**Correlations between the expression levels of signature genes in GC and immune infiltration using TIMER.** (**A**) AURKB, (**B**) BIRC5, (**C**) BUB1, (**D**) TPX2, (**E**) SST, and (**F**) GNG7. *P* < 0.05 was regarded as statistically significant. GC, gastric cancer; TIMER, Tumor Immune Estimation Resource; TPM, transcripts per million.

As one of the important components of the tumor microenvironment, immune cells play important roles in the progression and prognosis of GC [32, 33]. For an in-depth insight into the prognostic implication of the identified signature genes, the “Gene” module of TIMER was utilized to detect the associations between the expression of AURKB, BIRC5, BUB1, TPX2, GNG7, and SST and infiltration of immune cells in GC. Of note, the results revealed that BIRC5, BUB1, and TPX2 were positively correlated with tumor purity, whereas GNG7 and SST were negatively correlated with tumor purity (Figure 5). Regarding immune infiltration, AURKB expression was negatively correlated with B cells, CD8+ T cells, CD4+ T cells, macrophages, and dendritic cells (Figure 5A). BIRC5 expression was negatively correlated with the infiltration of B cells, CD4+ T cells, macrophages, and dendritic cells (Figure 5B). BUB1 expression was negatively correlated with the infiltration of all host immune cells, except for that of neutrophils (Figure 5C). TPX2 expression was negatively correlated with the infiltration of all immune cell types in GC (Figure 5D). Finally, SST expression was positively correlated with the infiltration of B cells, CD4+ T cells, and macrophages (Figure 5E), whereas GNG7 expression was positively correlated with the infiltration of all immune cells in GC (Figure 5F). These results suggest that the signature genes are closely associated with immune infiltration in GC and that they can be used as effective prognostic biomarkers for patients with GC.

### Correlation and diagnostic value analysis of signature genes

**Figure 6:**
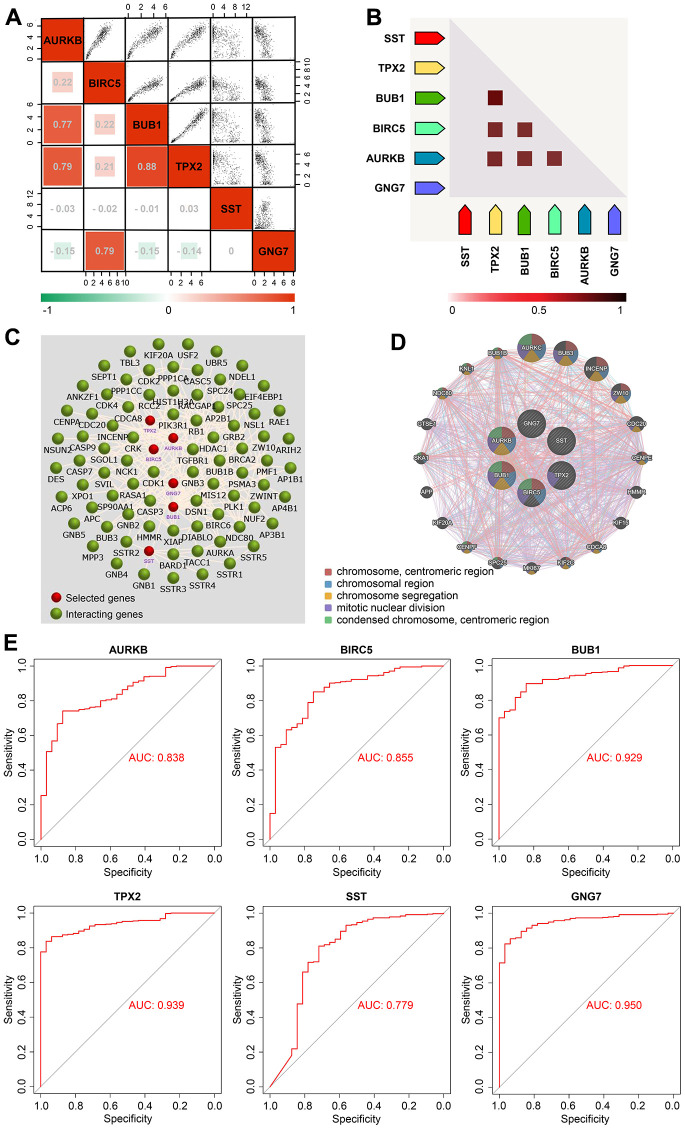
**Correlation and diagnostic value analyses of the signature genes in GC.** (**A**) Correlation heatmap of the six signature genes in GC presented using the GEPIA2 database. (**B**) Coexpression heatmap of the six signature genes in GC visualized via the STRING database. (**C**) Interaction network of the six signature genes and their related genes created using the FunRich software. (**D**) PPI network of the six signature genes and their coexpression genes obtained via GeneMANIA. (**E**) ROC curves of the six signature genes in the TCGA-STAD cohort constructed using the bioinformatics platform (http://www.bioinformatics.com.cn). AUC, area under curve; GC, gastric cancer; GEPIA, Gene Expression Profiling Interactive Analysis; PPI, protein–protein interaction; ROC, receiver operating characteristic; STAD, stomach adenocarcinoma; STRING, Search Tool for the Retrieval of Interacting Genes; and TCGA, The Cancer Genome Atlas.

Correlation analysis using GEPIA2 revealed that among the six signature genes, the upregulated genes (AURKB, BIRC5, BUB1, and TPX2) were largely negatively correlated with the downregulated genes (SST and GNG7) in GC (Figure 6A). Besides, the upregulated genes were positively correlated with each other, which was further confirmed via the coexpression heatmap obtained from the STRING database (Figure 6A, 6B). The interaction network of the signature genes and their related genes constructed using FunRich software revealed that these six genes might actively interact with each other (Figure 6C). Moreover, the PPI network of the signature genes and their coexpression genes constructed using the GeneMANIA database unveiled that these genes were predominantly enriched in the chromosomal centromeric region, chromosomal region, and chromosome segregation (Figure 6D). With respect to diagnostic value analysis, the study data showed that the area under the curve values of AURKB, BIRC5, BUB1, TPX2, SST, and GNG7 were 0.838, 0.855, 0.929, 0.939, 0.779, and 0.950, respectively (Figure 6E), indicating the relatively strong ability of the six signature genes to distinguish patients with GC from healthy subjects.

### GNG7 is downregulated in GC and correlated with tumor progression

**Figure 7:**
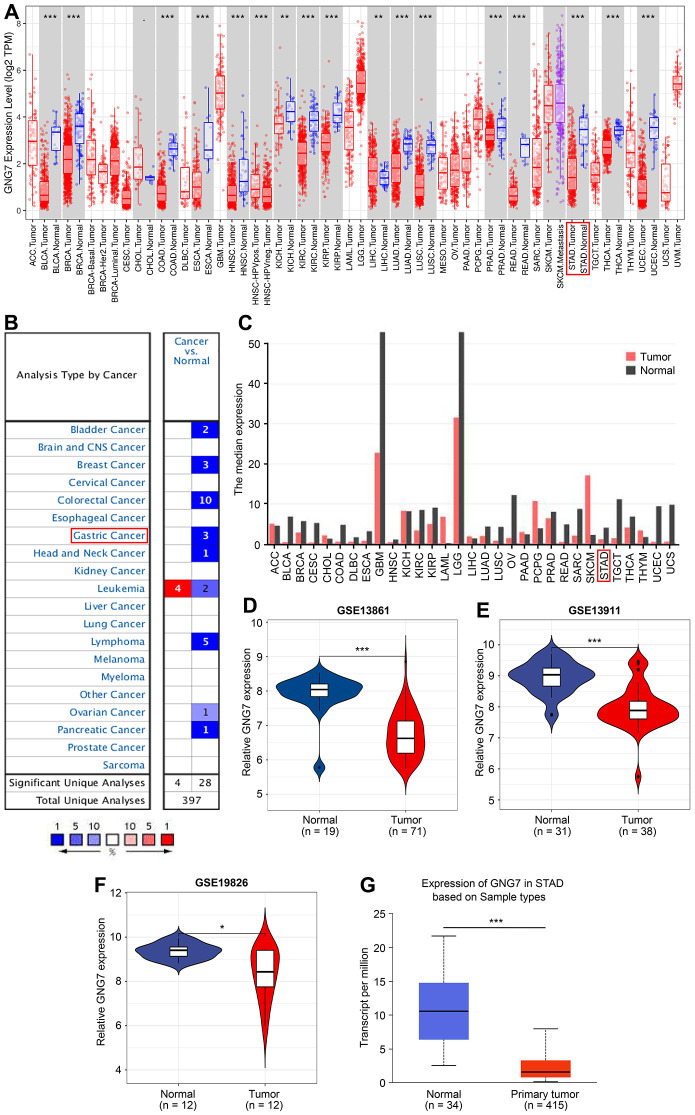
**Expression patterns of GNG7 in GC analyzed using bioinformatics tools.** (**A**) The expression of GNG7 in multiple human cancers was visualized using the TIMER database. (**B**) Expression of GNG7 in different human cancers was revealed using the Oncomine database. *P* < 1E-4 and |log_2_FC| > 2 were utilized as the statistic threshold. (**C**) Expression of GNG7 in multiple human cancers was analyzed via the GEPIA web tool. (**D**–**F**) Expression levels of GNG7 in GC and normal samples were derived from the GSE13861, GSE13911, and GSE19826 datasets. (**G**) Expression of GNG7 in STAD was detected using the UALCAN database. AML, acute myelogenous leukemia; FC, fold change; GC, gastric cancer; GEPIA, Gene Expression Profiling Interactive Analysis; STAD, stomach adenocarcinoma; and TIMER, Tumor Immune Estimation Resource. *P < 0.05, **P < 0.01, and ***P < 0.001 versus control.

To the best of our best knowledge, GNG7 has seldom been reported with GC in literature, but compared with the other signature genes, the present study results revealed that GNG7 had a higher diagnostic power for patients with GC. Therefore, GNG7 was selected as the target for subsequent analysis. The expression pattern of GNG7 in multiple human tumors was first analyzed using the TIMER database. The results revealed that compared with normal tissues, the expression level of GNG7 was significantly lower in several malignancies, including STAD, colon adenocarcinoma, rectum adenocarcinoma, and others (*P* < 0.01; Figure 7A). Consistent with this, data from the Oncomine and GEPIA tools also revealed that GNG7 was downregulated in diverse human cancers, including GC (Figure 7B, 7C). Furthermore, the mRNA expression level of GNG7 was remarkably decreased in GC compared with that in normal controls in the GSE13861 (*P* < 0.001; Figure 7D), GSE13911 (*P* < 0.01; Figure 7E) and GSE19826 (*P* < 0.05; Figure 7F) datasets and in the UALCAN database (*P* < 0.001; Figure 7G).

**Figure 8:**
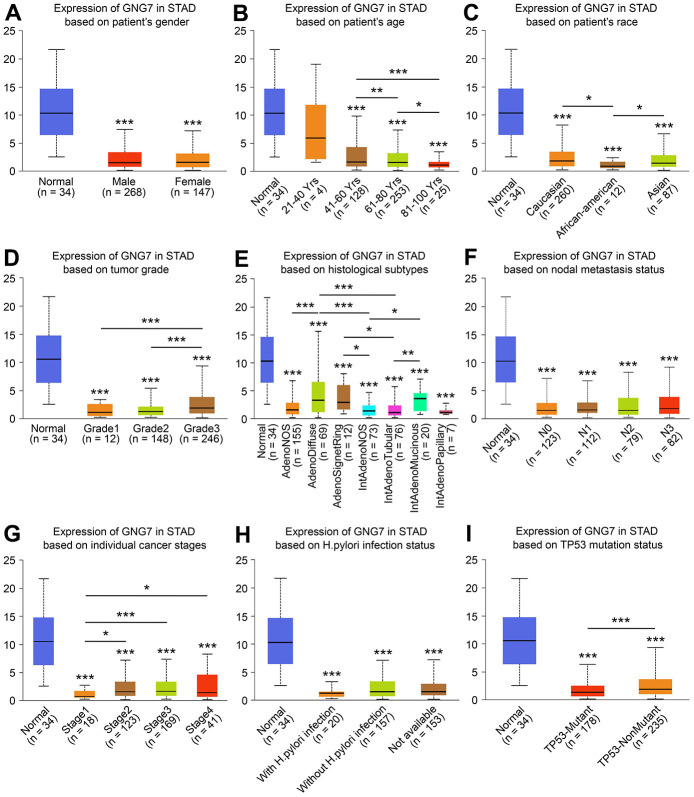
**Relationships between GNG7 expression and clinicopathological parameters.** Associations between the expression of GNG7 and sex (**A**), age (**B**), race (**C**), tumor grade (**D**), histological subtypes (**E**), nodal metastasis status (**F**), cancer stage (**G**), *Helicobacter pylori* infection status (**H**), and TP53 mutation status (**I**) of patients with STAD (UALCAN). STAD, stomach adenocarcinoma. *P < 0.05, **P < 0.01, and ***P < 0.001 versus control. (The asterisks above the error bar represent the comparison to the normal sample group, whereas the asterisks above the secondary line represent the comparison between groups indicated by the line).

To assess the clinicopathological significance of GNG7 in GC, the UALCAN database was searched, which revealed that the expression of GNG7 was correlated with the race, age, histological subtype, tumor grade, individual cancer stage, and TP53 mutation status of patients with GC (*P* < 0.05, Figure 8). However, the expression was not correlated with other clinicopathological parameters, including patient sex, nodal metastasis status, and *Helicobacter pylori* infection status (*P* > 0.05, Figure 8). Taken together, the results suggest that GNG7 is downregulated in GC and correlated with tumor progression.

### Enrichment analysis of GNG7-coexpressed genes and gene sets

**Figure 9:**
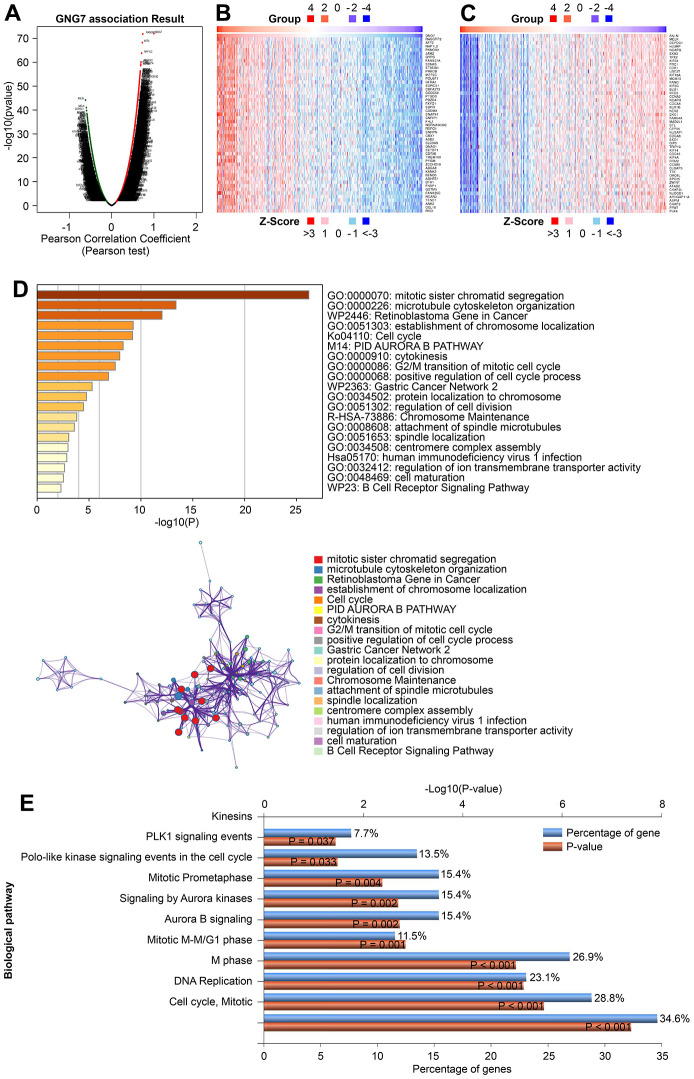
**Enrichment analysis of GNG7-coexpressed genes in GC.** (**A**) Volcano plot showing the correlations between GNG7 and genes differentially expressed in TCGA-STAD using Pearson’s test. (**B**, **C**) Heatmaps showing the top 50 genes positively and negatively correlated with GNG7 in TCGA-STAD. (**D**) Biological process enrichment analysis of GNG7-coexpressed genes in STAD performed using the Metascape database. (**E**) Biological pathway enrichment analysis of GNG7-coexpressed genes in STAD performed using the FunRich software. GC, gastric cancer; STAD, stomach adenocarcinoma.

**Figure 10:**
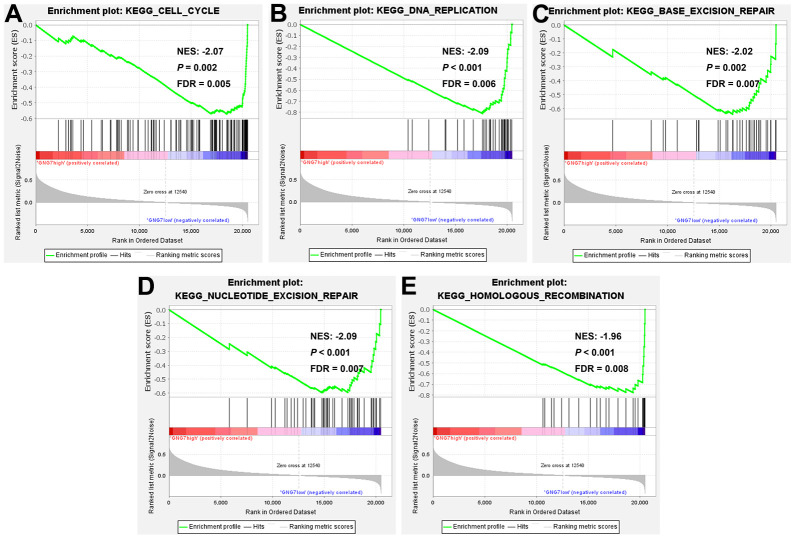
**Gene set enrichment analysis (GSEA).** KEGG pathway enrichment analysis of gene sets with lowly expressed GNG7 performed using TCGA-STAD data (**A**–**E**). FDR, false discovery rate; GSEA, gene set enrichment analysis; KEGG, Kyoto Encyclopedia of Genes and Genomes; NES, normalized enrichment score; and STAD, stomach adenocarcinoma.

To further explore the functional roles of GNG7 in GC, the LinkedOmics database was utilized to identify the genes coexpressed with GNG7. As illustrated in the volcano plot (Figure 9A), 8,229 genes (red dots) were positively correlated with GNG7, whereas 4,758 genes (green dots) were negatively correlated with GNG7. The top 50 genes that were positively or negatively correlated with GNG7 were visualized in heatmaps (Figure 9B, 9C). Enrichment analysis using the Metascape database revealed that the above 100 genes coexpressed with GNG7 were principally involved with mitotic sister chromatid segregation, microtubule cytoskeleton organization, the retinoblastoma gene in cancer, establishment of chromosome localization, and cell cycle (Figure 9D). Biological pathway analysis using Funrich software unveiled that the genes coexpressed with GNG7 were significantly enriched in cell cycle, DNA replication, M phase, mitotic M–M/G1 phase, and aurora B signaling (Figure 9E). Moreover, the results of GSEA further confirmed that the gene sets associated with the low expression of GNG7 in GC were enriched in cell cycle, DNA replication, base excision repair, nucleotide excision repair, and homologous recombination KEGG pathways (*P* < 0.01, Figure 10). Taken together, GNG7 may play important roles in the proliferation and cell cycle processes of GC cells.

### GNG7 inhibits cell growth by inducing cell cycle arrest and apoptosis in GC

**Figure 11:**
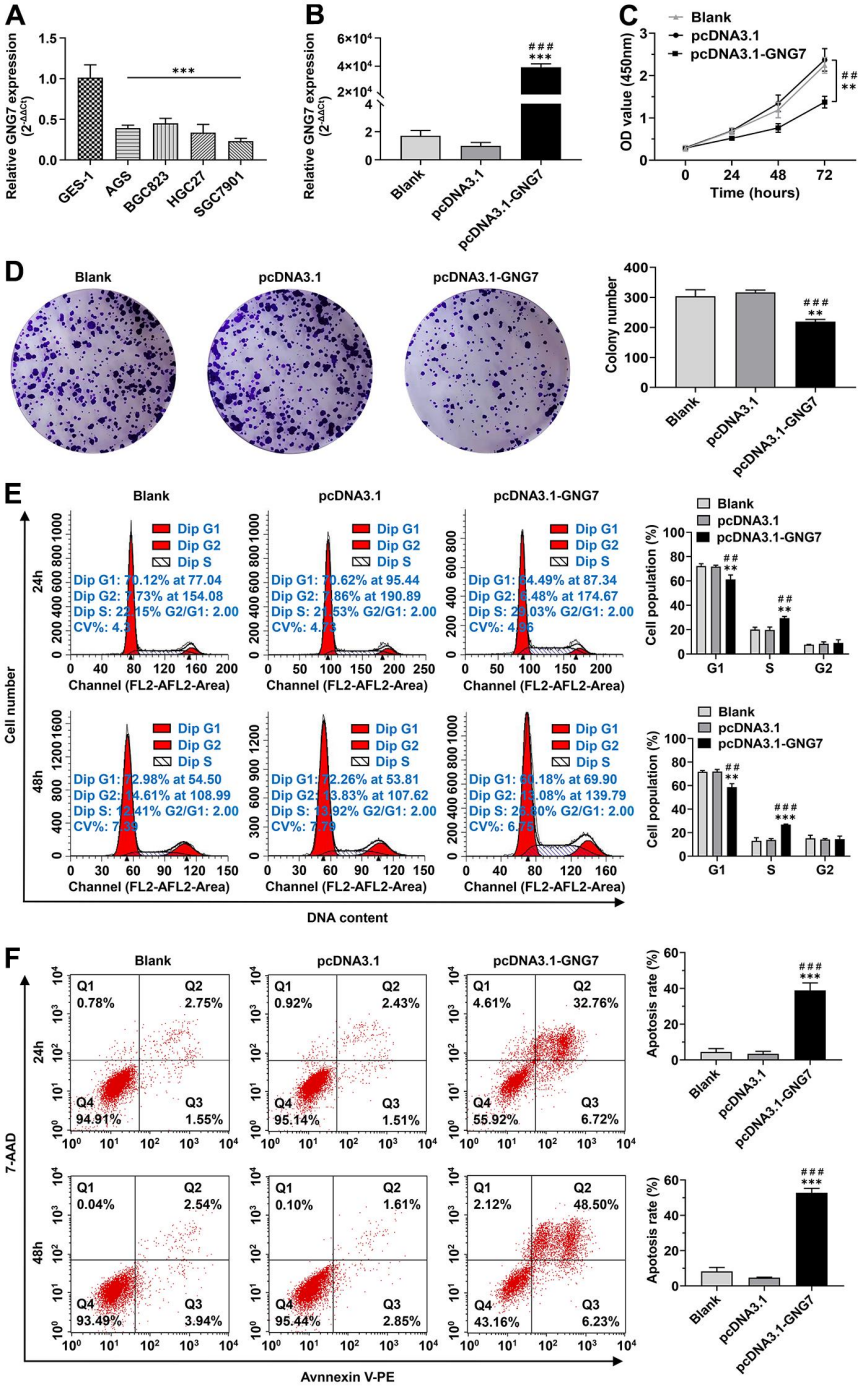
**Overexpression of GNG7 inhibited the proliferation and clone-formation abilities and induced cell cycle arrest and apoptosis in GC cells.** (**A**) GNG7 mRNA levels in GC cell lines (AGS, BGC823, HGC27 and SGC7901) compared to the normal epithelial cell line GES-1. (**B**) GNG7 mRNA levels in control and GNG7-overexpressing SGC7901 cells. (**C**, **D**) Proliferation and colony formation abilities of control and GNG7-overexpressing SGC7901 cells. (**E**) Cell cycle distribution in control and GNG7-overexpressing SGC7901 cells. (**F**) Cell apoptosis rate in control and GNG7-overexpressing SGC7901 cells. GC, gastric cancer. **P < 0.01 and ***P < 0.001 versus control or blank group; ^##^*P* < 0.01 and ^###^*P* < 0.001 versus pcDNA3.1 group.

The qRT-PCR assay revealed that the mRNA expression level of GNG7 was remarkably lower in four GC cell lines than in the normal gastric epithelial cell line GES-1 (*P* < 0.01; Figure 11A). To explore the potential roles of GNG7 in the malignant progression of GC, GNG7^low^ SGC7901 cells were selected for transfection with pcDNA-GNG7 plasmids for functional experiments. The GNG7 overexpression efficiency was quantified using qRT-PCR, and the results validated its upregulation in GNG7-overexpressing SGC7901 cells (*P* < 0.001; Figure 11B). Moreover, its overexpression observably elevated or depressed the top genes that were positively or negatively correlated with GNG7 (*P* < 0.05; Supplementary Figure 3). The CCK-8 and colony formation assays revealed that the overexpression of GNG7 markedly inhibited the cell proliferation and colony formation abilities of SGC7901 cells (*P* < 0.01; Figure 11C, 11D). The cell cycle assay showed that the overexpression of GNG7 could notably decrease the proportion of SGC7901 cells in the G1 phase and increase the proportion in the S phase (*P* < 0.01; Figure 11E). However, the proportion of cells in the G2 phase was not significantly different (*P* > 0.01; Figure 11E). The results of the apoptosis assay showed that the percentage of apoptotic cells in GNG7-overexpressing SGC7901 cells was significantly increased compared with that in the control cells (*P* < 0.001; Figure 11F). The evidence indicated that GNG7 inhibits the growth of GC cells by enhancing cell cycle arrest and inducing cell apoptosis.

## DISCUSSION

GC is one of the most prevalent and deadliest malignancies and the main cause of tumor-associated death worldwide [1]. Although tremendous advances have been made in the prevention and management of GC in recent decades, the prognosis and quality of life of patients diagnosed with GC remain dismal [5, 6]. The diagnostic abilities of traditional tumor markers such as CEA, CA 125, CA19-9 and CA 72-4 for GC are largely limited owing to their inadequate sensitivity or specificity [34, 35]. Furthermore, there is still a lack of ideal biological indicators to predict the progression and prognosis of GC and to be used as effective therapeutic targets in clinical practice [36]. Thus, the identification of novel biomarkers that play essential roles in the progression of GC, indicate treatment response, and act as targets for improving the prognosis of patients with GC is desperately needed.

In the present study, a total of 407 GC tissues and 64 normal tissues from the GSE65801 and TCGA-STAD datasets were included, which revealed 897 overlapping DEGs, including 392 downregulated and 505 upregulated DEGs. GO analysis showed that the upregulated DEGs were primarily enriched in extracellular matrix organization, extracellular structure organization, proteinaceous extracellular matrix, extracellular matrix, cytokine activity, and extracellular matrix structural constituent, whereas the downregulated DEGs were involved in digestion, chemical synaptic transmission, extracellular region, extracellular region part, gated channel activity, and ligand-gated channel activity. In addition, KEGG pathway analysis revealed that the upregulated DEGs were predominantly enriched in extracellular matrix–receptor interaction, cytokine–cytokine receptor interaction, and focal adhesion, whereas the downregulated DEGs were associated with gastric acid secretion, neuroactive ligand–receptor interaction, and chemical carcinogenesis. Following the PPI network and module analyses, the KEGG pathways enriched in the top modules of the constructed PPI networks were interpreted using the DAVID database. The results showed that the top three modules of the PPI network from the upregulated DEGs were enriched in cell cycle, extracellular matrix–receptor interaction, and phagosome, whereas the top three modules of the PPI network of the downregulated DEGs were correlated with cAMP signaling pathway, chemical carcinogenesis, and neuroactive ligand–receptor interaction.

Through in-depth analysis using three methods in the CytoHubba plug-in of Cytoscape, a total of 23 DEGs were identified as candidate hub genes, of which 20 (CDK1, CCNA2, AURKB, BUB1B, TOP2A, BIRC5, CCNB2, BUB1, CENPF, KIF2C, PBK, NCAPG, TPX2, KIF15, SST, CHGA, GNG7, NPY, GHRL, and CCKBR) were selected as hub genes for their expression patterns in GEPIA2, which is in accordance with our previous analysis of the identified DEGs and candidate hub genes. Because the associations between the expression levels of six hub genes (AURKB, BIRC5, BUB1, TPX2, GNG7, and SST) and overall survival of patients with GC were consistent with our speculation, they were chosen as prognostic signature genes. The involvement of the signature genes in the process of immune infiltration in GC was also confirmed. The correlation and coexpression analyses revealed that these signature genes were correlated and that they interacted with each other. Furthermore, all signature genes had good diagnostic efficiency in distinguishing GC specimens from normal specimens; among the genes, GNG7 displayed the highest diagnostic capability.

For a comprehensive understanding of the roles of the identified signature genes in the development and progression of GC, the relevant literature was reviewed. AURKB, also called AIM1 or AIK2, is an important member of the Aurora kinase family that participates in regulating the transition from the G2 phase to M phase during mitosis [37, 38]. Wang et al. found that AURKB was upregulated in GC and that the knockdown of AURKB inhibited the proliferation, invasion, migration, and cell cycle progression of GC cells in addition to inducing apoptosis [39]. Nie et al. revealed that AURKB could facilitate GC progression by activating CCND1, resulting in the poor overall survival of patients with GC [40]. BIRC5, also known as survivin, belongs to the inhibitor of apoptosis protein family; the proteins of this family play crucial roles in the processes of cell division and apoptosis suppression [41, 42]. Studies have shown an increased expression level of BIRC5 in GC, which was correlated with the depth of tumor invasion, distant metastasis, and TNM stage of GC [43–45]. As a member of the mitotic checkpoint family, BUB1 was upregulated in GC and correlated with the tumor histological subtype [46]. In addition, the increased expression level of BUB1 was further shown to be closely associated with tumor cell proliferation [47, 48]. TPX2 is a microtubule-related protein that is important in spindle formation and cell cycle progression [49, 50]. Tomii et al. unveiled that the elevated expression of TPX2 was correlated with lymph node metastasis, remote metastasis, and TNM stage of GC [51]. Meanwhile, Liang et al. demonstrated that the knockdown of TPX2 inhibited the proliferation, migration, and invasion of GC cells in addition to inducing cell cycle arrest and apoptosis [52]. SST, a cyclic polypeptide, is not only involved in the maintenance of internal environment homeostasis but also acts as a tumor suppressor in multiple cancers [53]. Chen et al. revealed that the knockdown of SST promoted the migration and invasion abilities of GC cells [54]; however, Wang et al. observed the opposite following SST overexpression in GC cells [55]. The G proteins are heterotrimers, comprising α, β, and γ subunits, which play important roles in transmembrane signal transduction [56, 57]. The G protein γ subunit has 12 members, and owns the ability to stabilize and localize G proteins to the cell membrane and mediate the signaling involved in cell growth [56, 58–61]. As a member of the G protein γ family, GNG7 was firstly cloned from bovine brain by Cali et al. in the year of 1992 [62]. Human GNG7 locates on chromosome 19p13.3, and predominantly expresses in some regions of brain such as striatum, hippocampus and neocortex [63–65]. In addition, GNG7 is reported to combine with Gα_olf_/Gβ_2_ and form a heterotrimeric complex, which participates in the neuro-protective response mediated by the A_2A_ adenosine or D1 dopamine receptor (D1R) [66–68]. Moreover, through ribozyme technology, studies also confirm the role of GNG7 in mediating the signal transduction between the β-adrenergic receptor and adenylyl cyclase via D1R in human embryonic kidney cells [69, 70]. Remarkably, GNG7 has been repeatedly shown to function as a tumor suppressor in many human cancers, including head and neck squamous cell carcinoma, esophageal cancer, breast cancer, pancreatic cancer, intrahepatic cholangiocarcinoma, clear cell renal cell carcinoma, and Hodgkin lymphoma [71–80]. However, the role of GNG7 in the malignant progression of GC remains undocumented.

According to bioinformatic analysis, GNG7 was significantly downregulated in GC. This finding was further validated via qRT-PCR. The results from the UALCAN database revealed that the expression of GNG7 was correlated with the race, age, histological subtypes, tumor grade, individual cancer stages, and TP53 mutation status of patients with GC. Of note, the enrichment analysis of GNG7-coexpressed genes and gene sets using data derived from the TCGA-STAD cohort attested that GNG7 was exceedingly implicated in the proliferation and cell cycle processes of GC cells. Many researchers have evaluated the effect of GNG7 in other cancer types. Liu et al. found that GNG7 overexpression shortened the G0/G1 phase and delayed the G2/M phase of cervical cancer cells [56]. Xu et al. showed that the knockdown of GNG7 promoted cell proliferation by shortening the G0/G1 phase and delaying the G2/M phase of cell cycle in clear cell renal cell carcinoma [78]. Shibata et al. revealed that GNG7 inhibited esophageal cancer cell growth by arresting the cell cycle at the G0/G1 phase [79]. Moreover, Mei et al. confirmed that GNG7 overexpression inhibited cell proliferation and induced apoptosis in breast cancer cells, whereas GNG7 knockdown showed reverse effects [75]. Consistent with these findings, the present study results revealed that compared with control cells, the proliferation and colony-formation abilities were decreased and the apoptotic levels were increased in GNG7-overexpressing SGC7901 cells. Furthermore, GNG7 overexpression shortened the G1 phase and delayed the S phase of SGC7901 cells without affecting the G2 phase, suggesting that GNG7 blocks cell cycle progression by inhibiting the progression of stage S to stage G2. Considering these findings, it could be concluded that GNG7 suppresses GC cell growth by arresting the cell cycle at the S phase and promoting cancer cell apoptosis.

## CONCLUSIONS

Taken together, a six-gene signature with a good ability to diagnose GC and predict the prognosis of patients with GC was identified using integrated bioinformatic analysis. The study revealed that GNG7 was downregulated in GC and inhibited cancer cell growth by blocking cell cycle progression and inducing apoptosis. Thus, GNG7 can be recommended as a novel biomarker and potential therapeutic target for patients with GC.

## Supplementary Material

Supplementary Figures
