# Supplementary material for: Identification of GNG7 as a novel biomarker and potential therapeutic target for gastric cancer via bioinformatic analysis and in vitro experiments
Source: Aging (Albany NY). 2023 Feb 24;15(5):1445–74. doi: 10.18632/aging.204545 (PMC10042700; doi:10.18632/aging.204545)
Supplement: Supplementary Figures [file aging-15-204545-s001.pdf]

## SUPPLEMENTARY FIGURES

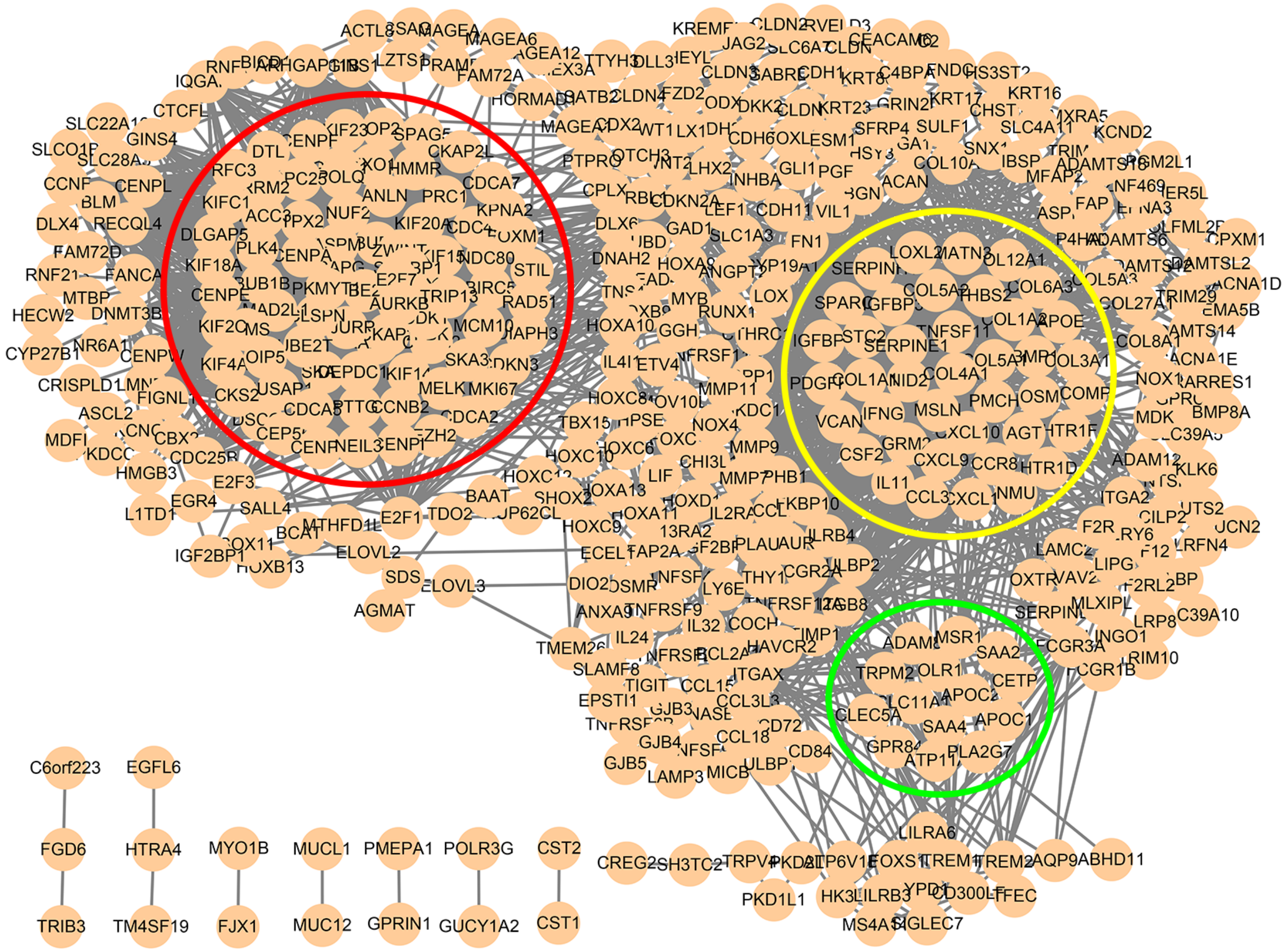


**Supplementary Figure 1. PPI network of the 505 upregulated intersecting differentially expressed genes (DEGs).** The red, yellow, and green circles represent the top three modules, respectively. DEGs, differentially expressed genes; PPI, protein–protein interaction.

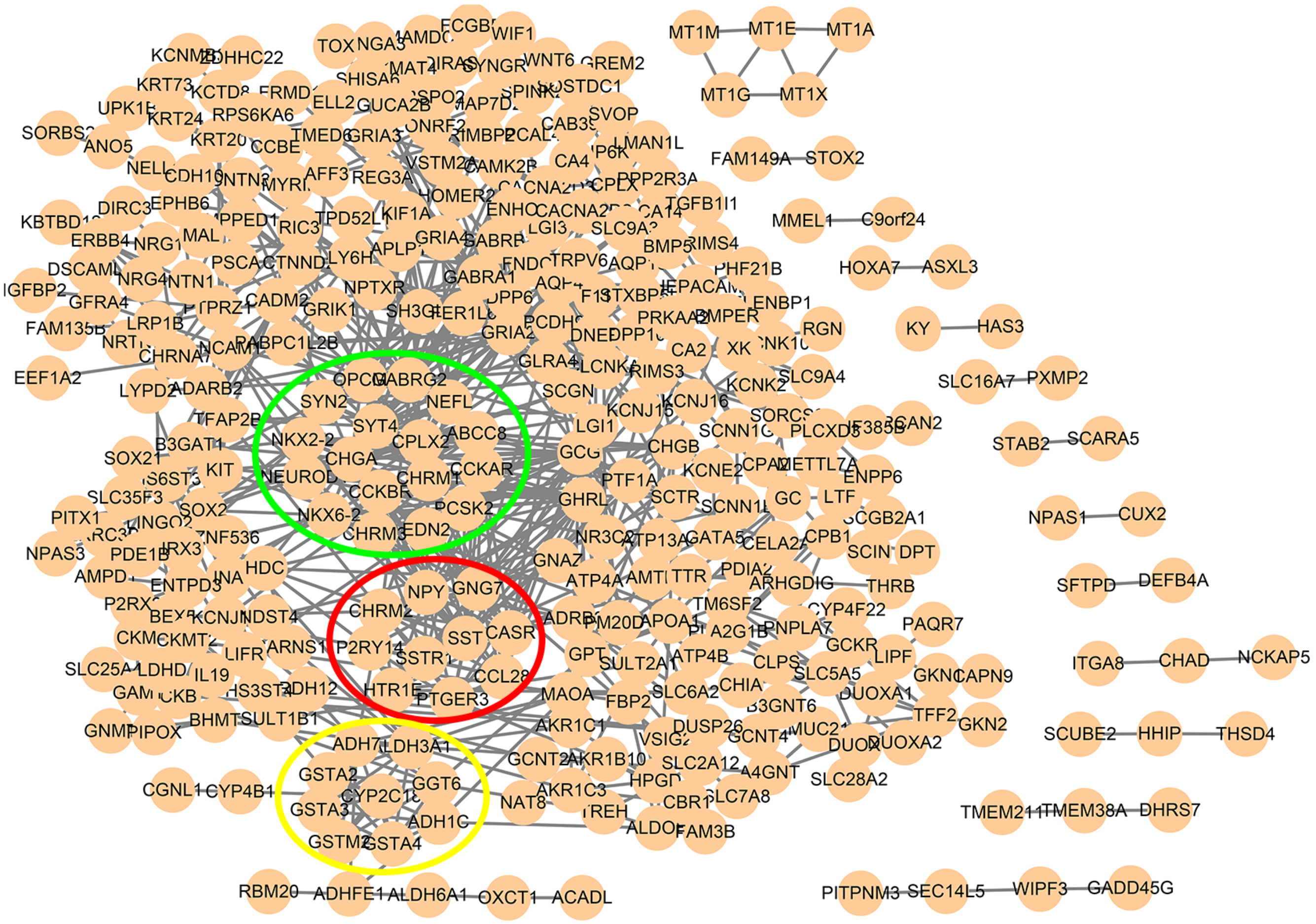


**Supplementary Figure 2. PPI network of the 392 downregulated intersecting differentially expressed genes (DEGs).** The red, yellow, and green circles represent the top three modules, respectively. DEGs, differentially expressed genes; PPI, protein–protein interaction.

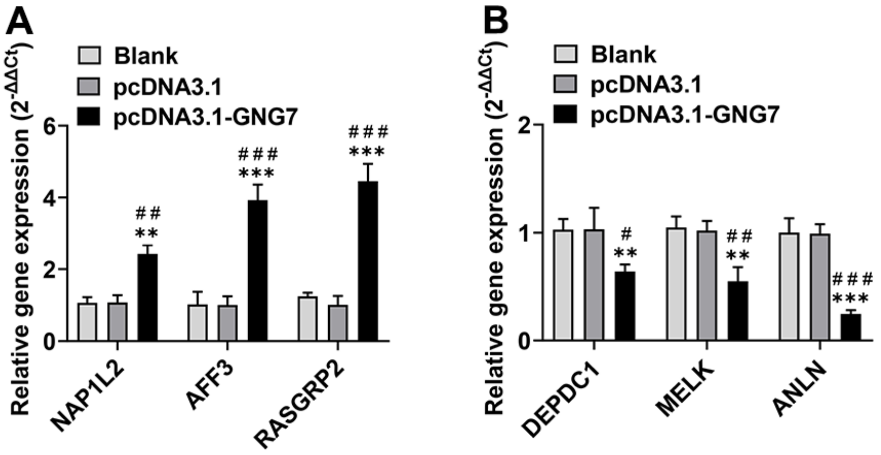


**Supplementary Figure 3. Overexpression of GNG7 regulated the expression of its coexpression genes in GC.** Expression levels of the top three positively (**A**) or negatively (**B**) correlated genes in control and GNG7-overexpressing SGC7901 cells determined by qRT-PCR. GC, gastric cancer; qRT-PCR, RNA extraction and quantitative reverse transcription-polymerase chain reaction. $^{**}P < 0.01$ and $^{***}P < 0.001$ versus control or blank group; $^{\#}P < 0.05$, $^{\#\#}P < 0.01$ and $^{\#\#\#}P < 0.001$ versus pcDNA3.1 group.
